# Role of Bacterial Cytoskeleton and Other Apparatuses in Cell Communication

**DOI:** 10.3389/fmolb.2020.00158

**Published:** 2020-07-16

**Authors:** Divya Singhi, Preeti Srivastava

**Affiliations:** Department of Biochemical Engineering and Biotechnology, Indian Institute of Technology Delhi, New Delhi, India

**Keywords:** MreB, membrane vesicles, curli, nanotube, tubular spinae 3

## Abstract

The bacterial cytoskeleton is crucial for sensing the external environment and plays a major role in cell to cell communication. There are several other apparatuses such as conjugation tubes, membrane vesicles, and nanotubes used by bacterial cells for communication. The present review article describes the various bacterial cytoskeletal proteins and other apparatuses, the physical structures they form and their role in sensing environmental stress. The implications of this cellular communication in pathogenicity are discussed.

## Introduction

In order to survive in the changing environmental conditions, microorganisms have evolved two broadly described complex communication systems- the contact-independent quorum sensing (QS) and the contact-dependent signaling mechanisms (Bassler and Losick, 2006; Blango and Mulvey, 2009). Cellular communications are usually mediated through synthesis, secretion and detection of signaling molecules commonly known as the inducers, which are released in the environment directly or through various cellular apparatuses (Kaprelyants and Kell, 1996). The mechanism of cell-cell communication that allows bacteria to share information about cell density and adjust gene expression accordingly through chemical signaling is known as QS (Miller and Bassler, 2001; Schertzer and Whiteley, 2011). As a part of a symbiotic relationship, QS was first observed in a bioluminescent bacterium *Vibrio fischeri* which lived on the light producing organ (photophore) of the bobtail squid (Bassler, 2002; Waters and Bassler, 2005). These interactions can be intra and/or inter-species or even inter-kingdoms and allowed co-existence of both the bacterium and its host. These communication systems are also the means through which the pathogens communicate and control their virulence traits. The phenomenon of QS has been extensively described in recent reviews (Papenfort and Bassler, 2016; Abisado et al., 2018; Mukherjee and Bassler, 2019).

The present review describes the role of bacterial cytoskeleton and contact dependent signaling through cellular apparatuses which play a major role in cellular communication. Bacteria have well-defined physical structures and cytoskeleton system which play key role in cellular communication.

**FIGURE 1:**
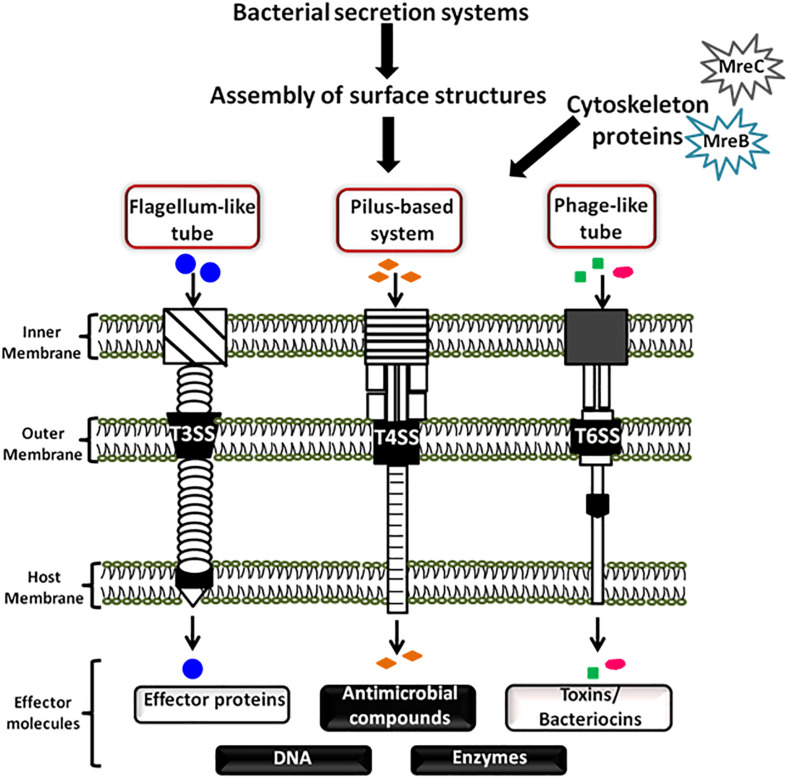
Schematic diagram showing bacterial cell communication mediated by surface appendages and cytoskeleton proteins. Bacteria have evolved two broad ways of communication systems- the contact-independent and the contact-dependent signaling mechanisms. The figure is a schematic showing cellular communication mediated by the bacterial cytoskeleton proteins and contact-dependent signaling. The cellular apparatuses which play a major role in cellular communication through contact-dependent signaling are assembled by the secretion systems. These appendages transfer the secreted effector molecules (proteins, DNA, antimicrobial compounds, toxins, and enzymes) by intra and/or inter-species or even inter-kingdom interactions.

As community behavior is highly complex, it can involve various genetic loci encoding extracellular factors and structures that promote surface sensing, cell-to-cell contact and surface colonization. These structures are usually the tubular cellular extensions such as curli, pili, flagella, and fimbriae, etc which help bacteria to communicate with their environment (Van Houdt and Michiels, 2005; Kline et al., 2009). These structures are often assembled with the help of secretion systems of the cell. In bacteria, till date there are nine major groups of bacterial secretion machinery reported from type I to type IX along with some additional categorized groups such as fimbrial chaperone-usher pathway (CU), Curli pathway, outer membrane vesicle secretion system (OMVSS), etc (Abby et al., 2016; Behzadi, 2019). These systems and pathways are critically important for the survival or death of the bacterial cells because they are involved in wide range of cellular activities including invasion, virulence, pathogenesis, genetic material exchange, immunological and biological interactions, antimicrobial resistance, colonization, and biofilm formation, etc (Abby et al., 2016; Behzadi, 2019). It has been reported that secretion systems assemble various surface structures that interact with both prokaryotic as well as eukaryotic target cells to deliver DNA or protein effectors to modulate cell physiology and growth (Hayes et al., 2010). A schematic representation showing assembly of bacterial surface appendages by the three secretion systems and how these appendages mediate cell-to-cell communication along with the help of cytoskeleton protein is shown in Figure 1. Based upon the mechanistic studies of these secretion systems, various bacterial cell surface structures have been identified that play a role in contact-mediated signaling.

**FIGURE 2:**
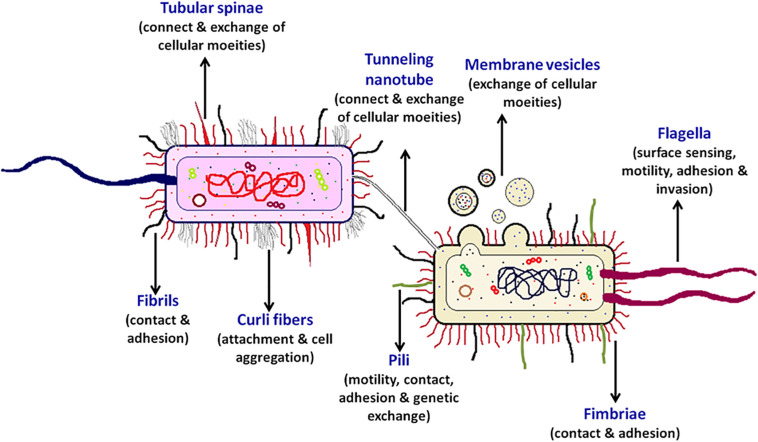
Bacterial cell-surface appendages. Various proteinaceous tubular or fibrous structures are found extending from the surface of bacterial cell wall. These structures help the bacterium in diverse functions such as in locomotion, adhesion, surface attachment, signaling, exchange of effector proteins, and genetic material, etc. Every cell surface appendage plays a specialized role and eventually helps in mediating bacterial communication. The strings like tubular structure flagellum, protruding outward from a bacterium is majorly known for providing motility, surface sensing, adhesion, and invasion. Pili helps in providing twitching motility to the bacterium, helps in adhesion and developing contacts for genetic exchange. The tunneling nanotubes are the tubular structures which help in exchange of cellular moieties in the adjacent cells. Whereas, the spherical shaped membrane vesicles help in exchange and delivery of various cellular moieties even to the far away cells. The curli fibers helps the bacterium in attachment and cell aggregation, the two filamentous surface appendages fimbriae and fibril help in contact and adhesion, the tubular spinae connect the cells and helps in the exchange of cellular substances.

Some of the bacterial surface structures that are directly or indirectly involved in cell signaling are shown in Figure 2 and described in Table 1.

**TABLE 1:** Bacterial surface appendages, composition and the list of microorganisms.

**S. No.**	**Surface appendages**	**Composition**	**Microorganisms**	**References**
1.	Curli fibers	Amyloid protein	*E. coli*, *Salmonella* spp., *Shigella*, *Citrobacter*, *Enterobacter* spp., *Pseudomonas* spp., *B. subtilis*, *S. coelicolor*, *S. aureus*, etc	Römling et al., 1998; Barnhart and Chapman, 2006; Nicastro and Tükel, 2019; Taglialegna et al., 2020
2.	Fibrils	Protein and polysaccharide	*M. xanthus*, *A. tumefaciens,S. typhimurium*, etc	Matthysse, 1983; Dworkin, 1999
3.	Fimbriae (SEF 14, SEF 21, Myf, pH6, F1, Type I, Type IV)	Protein (fimbrin)	*E. coli*, *K. pneumonia*, *Salmonella* spp., *Y. enterocolitica*, etc	Müller et al., 1991; Rehman et al., 2019
4.	Flagella	Globular protein (flagellin)	*E. coli, Vibrio* spp., *Pseudomonas* spp., *C. crescentus, S. eneterica*, *B*. *subtilis*, etc	Moens and Vanderleyden, 1996; Haiko and Westerlund-Wikström, 2013; Subramanian and Kearns, 2019
5.	Membrane vesicles (MVs, OMV, O-IMV)	Lipopolysaccharides, outer membrane proteins, phospholipids and periplasmic proteins	*P. aeruginosa, B. subtilis, Acinetobacter baumannii*, *Neisseria gonorrhoeae, Shewanella vesiculosa, Myxococcus xanthus, Flavobacterium* spp*., Vibrio* spp.	Deatherage et al., 2009; Turnbull et al., 2016
6.	Pili (Type I, Type IV)	Fibrous protein (pilin)	*E. coli, P. aeruginosa*, *S. enterica*, *V. cholera, A. tumefacians, Corynebacterium* spp., *Enterococcus* spp., *Ruminococcus* spp., *Streptococcus* spp., etc	Sauer et al., 2000; Kline et al., 2010; Lukaszczyk et al., 2019
7.	Tubular spinae	Protein (spinin)	*S. maritime, Rosebacter* spp., A*grobacterium* spp., *Chlorobium* spp., etc	Easterbrook and Coombs, 1976; Bayer and Easterbrook, 1991; Bernadac et al., 2012
8.	Tunneling nanotubes (TNTs/nanopods/nanowires)	Membranous segments/lipid bilayer/outer membrane and periplasmic extensions	*E. coli, B. subtilis*, *S. typhimurium, C. acetobutylicum, D. vulgaris, S. aureus*, etc	Dubey et al., 2016; Baidya et al., 2018

## Surface Appendages in Bacteria

### Curli Fibers

Curli are the highly aggregated extracellular amyloid fibers expressed by many Gram-negative enteric bacteria such as *E. coli* and *Salmonella* spp. (Chapman et al., 2002). They are the part of bacterial extracellular matrix through which the neighboring cells contact each other and form cell aggregates. With the studies conducted on *E. coli* curli, it was revealed that these fibers are produced either by a specialized secretion pathway commonly known as nucleation-precipitation mechanism or by well-defined Type VIII secretion system. These surface associated fibers are commonly 4–6 nm wide, varying in length up to several micrometers (Barnhart and Chapman, 2006). The structural and assembly components of these long amyloid (protein aggregates) fibers are encoded by seven curli-specific genes (*csg*) present in two different operons *csgBAC* and *csgDEFG*, respectively (Van Gerven et al., 2015). Curli plays an important role in cell aggregation and biofilm development, majorly during the attachment phase. The direct role of curli in causing pathogenesis has not been demonstrated but there are studies which suggest its role in infection process (attachment and invasion). Curli expressing bacterial cells attach better to the host cells as compared to those without it. Their invasion has been shown to result in activation of immune system causing host inflammatory response due to their interaction with host proteins (Barnhart and Chapman, 2006).

### Flagella

Being motile is one of the major advantages for the bacteria to adjust in adverse environmental stresses. Also, motility is considered as an important virulence factor of the pathogenic bacteria as it provides cell-to-surface contact required in the initial phase of pathogenicity. The most extensively studied mode of motility in bacteria is flagellum-mediated (Duan et al., 2013). Flagellum (plural-flagella) helps the bacterial cell to move toward the favorable environment. The structure of flagellum is well known consisting of three parts: the basal rotary motor, the hook joint and the helical propeller filament composed majorly of flagellin protein (Khan and Scholey, 2018). On the basis of number of flagella on the cell and their arrangements, motility and virulence of the bacteria dramatically varies. Monotrichous or single polar flagellum confers swimming motility to the bacteria in liquid (in *Vibrio* spp. and *Pseudomonas* spp. during planktonic growth) similar to the propeller on a boat and swarming on surfaces. Amphitrichous or one flagellum on each pole provides darting mobility to the bacteria (e.g., *Campylobacter jejuni*). In peritrichous arrangement or multiple flagella across the entire cell surface, run and tumble motility is adopted by the cell (Yang et al., 2016). This is observed in *Salmonella eneterica*, *E. coli* and *Bacillus subtilis*. Pathogenicity can be caused by bacteria only when it reaches the target site, colonize, invade the host tissue and proliferate. This makes motility an advantage for infectious bacteria. In the case of aflagellate mutants of various pathogens (*Borrelia burgdorferi*, *Campylobacter jejuni, Clostrodium chauvoei*, *Proteus mirabilis*, *Salmonella typhimurium*, etc.) it has been observed that their ability to cause a disease reduced drastically suggesting strong link between flagella and virulence (Penn and Luke, 1992; Moens and Vanderleyden, 1996). In addition to motility, flagella aid the bacteria even in rotating which is essential for adhesion in many cases. Flagella confers bacterial pathogenicity not only by providing propulsion or motility to the cell but also plays multiple functions such as surface sensing which is required for colonization through biofilm formation, adhesion or invasion to host epithelial cells, secretion of virulence factor, chemotaxis, and triggering a pro-inflammatory response of the host eukaryotic cells altering their immune system response mechanism (Duan et al., 2013). Besides all these, the sense of reaching a surface is also signaled by flagella. Once the bacteria reach a surface or come in contact with another cell, it attaches to the surface and provides signals to the cell about the surface contact which is actually the outcome of hindered rotation or motility (Kimkes and Heinemann, 2019). In *E. coli* and *P. mirabilis* with the variation in viscosity of their fluid environment, mechanical load varies which ultimately results in obstruction or inhibition of their flagellar rotation (Chawla et al., 2017). Similar results were observed with *B. subtilis*, *Caulobacter crescentus* and *Vibrio parahaemolyticus* in case of contact with surfaces, which provided evidence for the role of flagella in mechanism associated with surface-sensing and initiation of surface-dependent behavior (Lele et al., 2013; Gordon and Wang, 2019). This provides evidence that flagella are the main surface sensor in various bacteria and thus play an important role in pathogen-host interaction.

### Pili (pilus)

One of the mechanisms for microbial cell-to-cell interactions is through physical contact between the cells. These physical contacts or proximity between the cells are necessary for various function such as to assist the exchange of chemical signals and communicate, to develop multicellular structures. Moreover, for host-bacterium interactions and even for intracellular invasions by bacteria, physical contact becomes an essential requirement. One of the most important outcomes of physical interactions is the genetic exchange which leads to direct transfer of information from one cell to another cell. Such cell-to-cell interactions either between the cells of same species or others are often mediated by non-flagellar surface appendages known as pili.

Based upon the morphology and function, pili are classified into various sub-forms. There are long conjugative pili and short adhesive or attachment pili (Kuehn, 1997). Conjugative (F or sex) pili facilitate the transfer of genetic material between two bacterial cells. The two most extensively characterized pili of Gram-negative bacteria are Type I and Type IV pili. These pili are thin (2–8 nm diameters), several micrometers long and are non-covalently homopolymerized with their pilin subunits. The type I pili are common in the family Enterobacteriaceae and pathogenic *E. coli* strains. Apart from imparting adherence properties to the bacteria, they also play role in biofilm formation. The type IV pili are found in large variety of Gram-negative bacteria such as *Neisseria gonorrhoeae*, *Neisseria meningitides*, *Pseudomonas aeruginosa*, *Salmonella enterica*, *Vibrio cholerae*, etc (Craig et al., 2004). These pili besides playing key role in host cell adhesion, phage transduction, DNA uptake during transformation, most importantly provide “twitching motility” to the bacteria. The ability of type IV pili structures to undergo repetitive cycles of extension and retraction supports their pivotal roles in adhesion, natural transformation and motility (Adams et al., 2019). Motility is driven by interaction (polymerization) and retraction (depolymerization) of the major pilin subunit (PilA) in the presence of two cytoplasmic ATPase, PilF/B and PilT, respectively (Proft and Baker, 2009).

The pili observed in *Agrobacterium tumefaciens* are found to be similar to bacterial conjugative pili on the basis of their assembly and function but are morphologically distinct. These pili are observed on the surface of bacterium only under the induced expression of *vir* genes. Homologs of *vir* genes are even observed in bacterial species of *Bordetella* and *Helicobacter* in their toxin export systems. The adhesive pili are the general characteristic of pathogenic bacteria and are involved in initial adhesion of the bacterium to the host cells.

Pili are even detected on the surfaces of Gram-positive bacteria. There are various reports on characterization of pili in Gram-positive pathogenic bacteria such as *Clostridia*, *Corynebacterium*, *Enterococcus*, *Ruminococcus*, and *Streptococcus* (Telford et al., 2006). Apart from these, pili are also reported in various members of extensively studied Gram-positive Lactic acid bacteria (LAB) belonging to genera *Lactococcus* and *Lactobacillus*. Pili appendages aid in adhesion of these probiotic strains to intestinal epithelial cells and thus provide significant contribution in exerting the response of host immune system (Chapot-Chartier and Kulakauskas, 2014).

Broadly, two types of pili are identified in Gram-positive bacteria by electron microscopy. One form is of the thin (1–2 nm diameters) and short rods (70–500 nm length) whereas the other form is comparatively thicker (3–10 nm diameters), longer (0.3–3 μm length) and more flexible. The main building blocks of these pili are the subunits of pilin (pilus protein) present in multiple copies along with some minor pilus proteins or accessory pilins to form pilus shaft. These subunits are covalently connected and are anchored with the aid of sortase enzyme to the cell wall. The pili reported in the Gram-positive pathogens such as *Corynebacterium diphtheriae*, *Streptococcus pneumoniae* (pili detected in some strains not all), *Streptococcus pyogenes*, *Mycobacterium tuberculosis* and *Actinomyces naeslundii* are used as one of the most powerful means of cellular attachment and colonization (Proft and Baker, 2009).

It has been reported that various pathogenic bacteria, for instance some members belonging to genera *Pseudomonas* and *Xanthomonas* possess genes for hypersensitive reaction and pathogenicity (*hrp*). These *hrp* genes (mainly *hrpS* and *hrpC*) produces filamentous surface appendage known as Hrp pilus, responsible for bacterial entry into targeted host cell. The main structural protein of Hrp pilus is HrpA protein and in *hrpA* mutant, the bacterium becomes incapable of forming Hrp pilus and thus losses its ability to cause disease in plant (Roine et al., 1997). Similar to this, formation of *virB*-dependent pilus is reported in *A. tumefaciens* for transfer of T-DNA into plant cells (He, 1996). These reports suggest formation of surface appendages as a widespread attribute of both plant and animal pathogenic bacteria for causing infection (Dangl, 2013). Moreover, for surface-associated *P. aeruginosa*, pilus motors (type-IV pili) are found to be involved in mechanosensing. Mechanical tension generated either by inhibiting the retraction of type-IV pili motors or due to shearing and friction associated with the twitching motility and surface adhesion of the bacterium acted as the signals for biological responses. As a resultant, cAMP-dependent upregulation of virulence and an increase in the production of c-di-GMP involved in biofilm formation is observed in the bacterium (Rodesney et al., 2017). Furthermore, the crucial role of type IV pili has also been observed in *N. gonorrhoeae*. It has been reported that attachment of bacteria to the host cell and cell-cell interaction driven assembly of microcolonies on the surfaces are primarily mediated by type IV pili (Pönisch et al., 2017). In *Thermus thermophilus*, with the help of cryo-electron microscopy and mass spectrometry it is revealed that the bacterium possesses two different types of type IV pili- wide and narrow. Both not only differ on the basis of protein composition and structure but have different functions as well. The wide pili composed of the PilA4 pilin protein is majorly required by the bacterium during DNA uptake (natural transformation) whereas the narrow pili composed of the PilA5 pilin protein is required for the twitching motility (Neuhaus et al., 2020). Moreover, in *Geobacter sulfurreducens* contact-dependent mechanism of communication using pili as nanowires has been observed. It has been demonstrated that pili serves as the electric conduits for electron transfer in *G. sulfurreducens* fuel cells and on Fe (III) oxide surfaces to form biofilms (Reguera et al., 2007).

In general, DNA-uptake by pili can be studied in two steps; first step includes mechanistic possibilities of DNA-binding to the pili and second step could be the DNA path through cell’s initial barriers. Various mechanistic hypotheses for the same have been given but all vary between different bacteria (Piepenbrink, 2019).

### Tunneling Nanotubes/Intercellular Nanotubes

The tunneling nanotube (TNTs) connects bacteria of the same or different species and enables transfer of intra and intercellular contents (Marzo et al., 2012). Studies done on *B. subtilis*, *Staphylococcus aureus* and *E. coli* proposed TNTs as one of the major form of bacterial communication method for exchange of cellular molecules between and within the species (Dubey and Ben-Yehuda, 2011). Bacterial nanotubes are categorized into two basic forms: (1) The thin nanotubes that connect the nearby neighboring cells and (2) thick nanotubes connecting the distal cells. With the help of these TNTs, cell bridges communication with neighboring bacteria for exchange of cytoplasmic constituents, transmission of DNA and plasmids and also to acquire non-hereditary resistance to antibiotics from the nearby cells (Dubey and Ben-Yehuda, 2011). Interestingly, nanotube connections have been demonstrated in various other bacterial species as well. Under nutritional stress, the induced cell-to-cell interactions through nanotubular structures resulted in bidirectional exchange between Gram-positive bacterium *Clostridium acetobutylicum* and Gram-negative bacterium *Desulfovibrio vulgaris* (Benomar et al., 2015). Likewise, *Acinetobacter baylyi* and *E. coli* showed connection through membrane-derived nanotubes for exchange of nutrients (Pande et al., 2015). An intermediate structure (outer membrane tubes) between nanotube and chain-like structure has also been reported in *Myxococcus xanthus* for exchange of cellular moieties on cell-cell contact (Wei et al., 2014). Thus, nanotube connections have shown to help in distributing metabolic functions across the various microbial communities. Moreover such tubular connections are also been observed between *S. typhimurium* cells and its host eukaryotic cells resulting in a host-pathogen interaction (Galkina et al., 2011).

### Membrane Vesicles

Many bacteria release spherical, extracellular vesicles packaged with specific molecules involved in diverse functions. These membrane vesicles (MVs) have similar composition to that of the outer membrane consisting of lipopolysaccharides (LPS), outer membrane proteins, phospholipids and even some periplasmic proteins. In most cases, vesicles have only one membrane but two membranes i.e., outer and inner membrane vesicles (O-IMVs) are also observed in few bacterial species such as *Acinetobacter baumannii*, *N. gonorrhoeae*, *P. aeruginosa* and *Shewanella vesiculosa* (Pérez-Cruz et al., 2015).

The formation of MVs was earlier believed to take place by controlled blebbing of the outer membrane. Both Gram-negative and Gram-positive bacteria have different cell wall structure, it is likely that they posses different mechanisms for MVs formation (Toyofuku, 2019). Extensively studied Gram-negative bacterium *P. aeruginosa* demonstrated the formation of MVs through explosive cell lysis (Turnbull et al., 2016). However, Gram-positive bacterium such as *Bacillus subtilis* produces MVs through bubbling cell death. *Bacillus* cells did not explode rather died (ghost cells) but retained their cell morphology while releasing MVs (Toyofuku et al., 2019). Variation to these synthesis processes is the genesis of membrane tubes from the outer membrane as an intermediate before vesicle formation. An abiotic morphogenic process known as pearling transform these unstable intermediate membrane tubes into stabilized chains of interconnected vesicles. These chains of interconnected vesicles enlarge the cell surface of bacteria resulting into increased surface enzymes per cell volume (Bar-Ziv and Moses, 1994). These vesicle chains are well reported in various species of Flavobacteria such as *F. columnare*, *F. psychrophilum* and also in strain Hel3_A1_48 of a marine flavobacterium (Fischer et al., 2019). Apart from these, *Francisella novicida*, *Myxococcus xanthus*, and *Shewanella oneidensis* also showed chains of vesicles on their cell surfaces (Subramanian et al., 2018; Fischer et al., 2019).

These released membrane vesicles form one of the ways implied for prokaryotic communication. The packaged MVs travel and fuse with distant cells and thus facilitate exchange of various cellular molecules such as those involved in the process of QS, transfer of factors responsible for development of antimicrobial resistance, delivery of toxins, and even exchange of the genetic material (Dubey and Ben-Yehuda, 2011). MVs mediate communications between interspecies and also between different cells of interkingdom (Mashburn-Warren and Whiteley, 2006). Recent researches have shown that MVs fusion, their transmission to specific target cells in microbial and host-microbial interactions is self-guided. MVs have been demonstrated as the carrier of various signaling molecules involved in bacterial cell-to-cell communication. In pathogens like *P. aeruginosa*, MVs transport the interbacterial signaling molecule *pseudomonas* quinolone signal; PQS (2-heptyl-3-hydroxy-4-quinolone) to the bacterial population for dealing with the hostile environmental conditions (Häussler and Becker, 2008). Similarly, in marine pathogen *Vibrio harveyi*, outer membrane vesicles (OMVs) package the long-chain amino-ketone CAI-1 QS signaling molecule triggering the QS phenotype not only in CAI-1 non-producing *V. harveyi* but also in *V. cholerae* cells (Brameyer et al., 2018). Moreover, the coral-associated pathogen, bacterium *Vibrio shilonii* also releases OMVs containing N-acyl homoserine lactones (AHLs) signaling molecules, alkaline phosphatase, chitinase, and lipase (Li et al., 2016). Furthermore, in the soil bacterium *Paracoccus denitrificans* PD1222, the long-chain AHLs (C16 - N-(hexadecanoyl)- L-homoserine lactone) associated with cell-to-cell communication are majorly released in the population through MVs (Toyofuku et al., 2017).

### Tubular Spinae

The long, hollow, and tubular appendages known as spinae have been reported in various Gram-negative bacteria. Spinae are non-prosthecate (echinuliform) appendages which do not have any connection with the cytoplasm. They are about 3 μm in length, 50–70 nm in diameter and observed randomly on the cell surface (Kim, 2017). In Gram-negative pseudomonad *Spinomonas maritime*, production of long tubular surface appendages (spinae) is controlled by various growth parameters such as osmolarity, temperature and pH. It has been observed that at slightly elevated temperature of about 34°C with pH 7.4 and relatively low osmolarity of the growth medium, highest yield of spinae was obtained whereas no or very less number of spinae were observed at lower temperature, pH, and higher salt concentration (Easterbrook and Sperker, 1982). Apart from playing major role in cell protection from protozoan predators and cell sedimentation, there are reports which suggest spinae as long-distance cell-to-cell connectors. Examination of the *S. maritime* cells grown in low osmolarity medium through scanning and transmission electron microscopy showed various cells connected to each other through tubular spinae over distances of several micrometers (Bayer and Easterbrook, 1991). These surface appendages apart from permitting cell-to-cell signal exchange could also be a way of uniting similar or single type of cells in the present diverse forms of multicellular organisms.

### Fibrils

Another mechanism of cell-to-cell interaction is mediated through extracellular appendages known as fibrils. These appendages are reported in various bacteria but have been extensively studied only in the Gram-negative myxobacterium *Myxococcus xanthus* (Dworkin, 1999). They are filamentous organelles, 15–30 nm in diameter composed of equal amounts of polysaccharides and proteins. Fibrils are differentiated from pili based upon size as they are thicker and longer as compared to pili. They may be arranged either densely as clumps or tufts together or even can be sparsely distributed all over the bacterial cell surface. Similar to pili, fibrils are also necessary for the social behavior of the cells. They assist in maintaining the physical contact between the cells and even in between the cells and their substratum. Apart from this, there are various other examples of fibrils, morphologically similar to those described for *M. xanthus*. Fibrils also serve an important function of attaching the bacterial cell to their host targets as seen in the case of cellulose fibrils synthesized by the *Agrobacterium tumefaciens* (Matthysse, 1983). The fibrils thus synthesized anchor the bacteria to host cells and aid in production of crown gall tumors. Similarly, the short, stubby fibrillar appendages formed due to the contact between *S. typhimurium* and cultured epithelial cells have been shown to be necessary for internalization of the bacteria. Another important example is of an extracellular filamentous appendage haemagglutinin of the virulent *Bordetella* which in conjunction with the pertussis toxin causes pathogenicity.

### Fimbriae

Adhesion to the host cell is considered as the initial and one of the most crucial steps in pathogenesis. Functionally similar to fibril, another potential adhesin which mediates attachment of bacterial cell to the host receptor molecule is fimbriae. They are proteinaceous filamentous surface appendages consisting of helically arranged fimbrin (protein) monomers (Müller et al., 1991). Fimbriae though functionally seem similar to fibrils but are ultrastructurally as well as biochemically different entities. Fimbriae structures ranges in between 0.5 and 10 μm length and 2–8 nm width whereas fibrils lengths vary in different strains (Handley, 1990; Rehman et al., 2019). Presence of fimbriae in some cases provides strong indication toward bacterial virulence. They facilitate adhesion and are also involved in bacterial aggregation, colonization and biofilm formation (Müller et al., 1991). In host, these extracellular bacterial appendages play important role in interaction with macrophages and intestinal persistence. Initially, based upon their morphology and hemagglutination patterns (ability of the mannose monosaccharide to inhibit the adhesion of fimbriae to erythrocytes i.e., mannose sensitive or mannose resistant) fimbriae were categorized into 7 types ranging from Type I to VI and Type F (Clegg and Gerlach, 1987). These surface appendages types are common to various bacteria and have been extensively studied in various members of Enterobacteriaceae family such as in *E. coli*, *Klebsiella pneumonia*, *Salmonella* etc. But later, with the help of serological tests, genetic relatedness of fimbrial antigens was identified and fimbriae were further classified into 3 different types based upon their assembly pathways. The three assembly pathways which lead to multiple types of fimbriae are chaperon–usher (CU), nucleation–precipitation (N/P), and Type IV fimbriae (Rehman et al., 2019). SEF 14 and SEF 21 are the two comprehensively studied Type I fimbriae of *Salmonella enteritidis* (Müller et al., 1991). Thus, affinity provided by fimbriae to bind with host cell receptors helps in host-pathogen interaction and cause severe disease and infections like salmonellosis.

These surface appendages help in contact-dependent signaling, mechanosensing or inhibition of the receiver cell.

### Mechanism of Contact-Dependent Cellular Communication

The highly specialized secretion systems of bacteria secrete a variety of molecules including proteins and DNA which have significant role in bacterial communication. Based upon the type of secretion system, these secreted molecules have three possible outcomes, first is either they remain anchored to the outer membrane of the producer cell or second they get released into the extracellular space or third they are directly injected into the targeted bacterial or eukaryotic cell (Costa et al., 2015).

The type III, IV, V, and VI secretion systems (T3SSs, T4SSs, T5SSs, and T6SSs) provide important example of contact-sensor mechanism. Out of these systems, the effector molecules from T3SS, T4SS, and T6SS generally crosses all the three phospholipid membranes (two of producer bacterial cell and one of the hosts) and reaches the cytosol of host with the help of assembled surface structure (Figure 1). The T4SSs are pili-based secretion systems which majorly require cell-to-cell contact between the emitter and receiver cells. This contact is mediated by the cell surface adhesins and/or pili structures. In Gram-negative pathogens, it has been reported that the pili of T4SS interacts with various proteins of host cell surface (Hayes et al., 2010). Various other well reported pili such as the F-pilus from *E. coli* serve as a gripping hook that elongates and retracts from the cell surface to bring both donor and the recipient cell together for direct cell-to-cell interaction. Similarly, the P-pili of the conjugation systems act as the adhesive structure for binding together the mating cells. Moreover, in *A. tumefaciens* it has been shown that the primary component of the T-pilus VirB2 pilin protein is responsible for cell-cell interactions, assisted along with the VirB5 protein localized at the tip of the T-pilus (Aly and Baron, 2007; Backert et al., 2008).

In addition to this, the type III secretion systems (T3SSs) of various Gram-negative pathogens report the use of flagellum-like apparatus known as injectisome (needle complex) for the delivery of effector proteins to the target host cells (Cornelis, 2006). This apparatus has a similar structural organization to flagella and also shares amino acid sequences homology with several proteins (Hayes et al., 2010). Moreover, the component proteins (FlhA, FlhB, FliO, FliP, FliQ, FliR FliH, FliI, and FliJ) of the flagellar export apparatus shares considerable sequence similarities with the pathogenic bacteria possessing type III secretion system (Minamino, 2014).

Likewise, studies on *Campylobacter jejuni* report the importance of cell surface appendages in developing the contact with host cells evading their defense systems. It has been shown that in order to possess antigenic diversity and provide a competitive advantage to *C. jejuni*, the surface structures are subjected to various modifications such as *O-* and *N*-linked glycosylation of the flagella filament. These flagellar modifications ultimately affect the pathogen- host interactions. Thus flagella are important for locomotion as well as for the pathogenesis of the bacterium (Cullen et al., 2012). Interestingly, another important example of bacterial cross-talk has been reported in *S. typhimurium*. The bacterium after sensing the signals from host assembles the surface appendages for further signaling. These surface-associated appendages help the bacterium in developing contact with the host cell to initiate bacterial uptake and thus demonstrates a significant example of two-way biochemical signaling for the adaptation of a pathogen by its host (Ginocchio et al., 1994).

**FIGURE 3:**
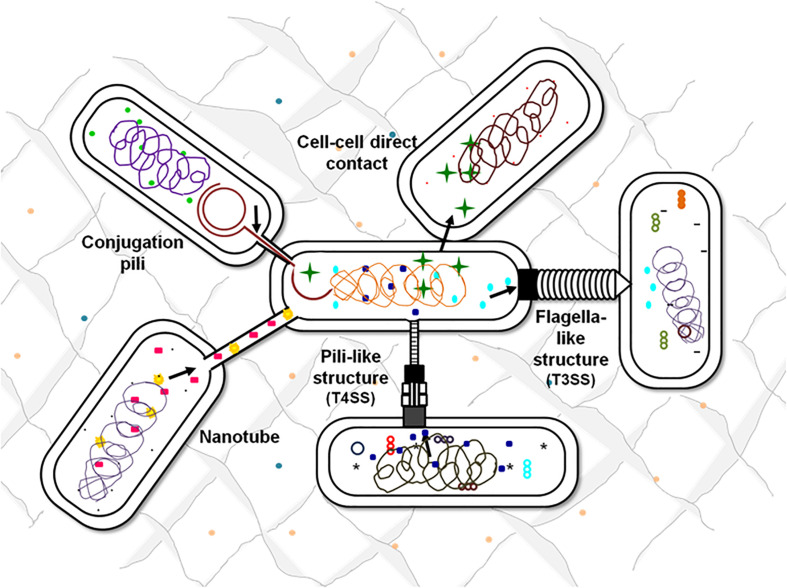
Contact-dependent signaling mechanisms. Effector molecules can be transferred from one cell to another using contact-mediated signaling methods. Exchange of metabolites can be either through the surface appendages or can be even by direct surface contact. Flagella-like structures assembled by T3SSs, pili-based structures by T4SSs, intercellular nanotubes, and conjugation pili are the appendages-based methods that facilitate the exchange of cellular contents.

There are multiple contact-dependent mechanisms by which bacteria can communicate; the major ones are depicted in Figure 3. Another example is of the syntrophic relationship between *Pelotomaculum thermopropionicum* and *Methanothermobacter thermautotrophicus.* The bacterium *P. thermopropionicum* liberates hydrogen gas by fermentation and *M. thermautotrophicus* is a methanogen that uses H_2_ to reduce CO_2_ (Ishii et al., 2005). The flagellum expressed by the *P. thermopropionicum* specifically bind to *M. thermautotrophicus* to trap it within range for H_2_ diffusion and enhance methanogenesis (Shimoyama et al., 2009; Hayes et al., 2010). In case of *M. xanthus*, the cell-cell signaling for the formation of fruiting bodies within which they sporulate is observed through direct physical interactions. A contact-dependent cue known as C-signal is exchanged when two cells comes in contact end-to-end (Kaiser, 2004; Visick and Fuqua, 205). Whereas, the other mechanisms involve cell surface appendages which develops the contact between two adjacent for even between two far-away cells to further mediate signaling. Therefore, it is observed that every cell surface appendage plays a specialized role in developing bacterial communication. The Curli fibers and type IV pili helps in recognition, adherence and even in invasion into the target cells whereas have no role in transport (Fronzes et al., 2008). In contrast to this, the tunneling nanotubes, tubular spinae connect the cell, and transport signaling molecules.

### Contact-Dependent Inhibition

In order to survive in the vast microbial community, bacteria exhibit various cooperative and competitive interaction mechanisms. One such contact-based competitive mechanism is Contact-dependent growth inhibition (CDI). CDI systems are evolved in bacteria as a mechanism to inhibit growth or kill the neighboring outcompeting cells. To execute its operation, CDI system requires a direct contact between the producer and the targeted cell. These systems deliver polymorphic proteinaceous toxins into the cytoplasm of adjacent competitors unless they produce a corresponding antidote protein. Thus, CDI system also mediates cooperative communication between the cells which produce identical toxin-antitoxin pairs (Garcia et al., 2016; Roussin et al., 2019).

The most widely studied contact-dependent growth inhibition systems present in various bacterial pathogens comprises of Cdi toxins (CdiA and CdiB). Mostly, the sec complex of the type V secretion system (T5SS) is used for translocation and anchoring of the CdiA and CdiB toxin from inner to the outer membrane of the producer cell and then directly deliver to the target cell. CdiA protein also promotes cell-cell adhesion needed for contact-dependent inhibition (García-Bayona et al., 2017). In addition to T5SS structures, the surface-associated appendages of T6SS and T7SS also deliver toxin directly to the adjacent cells (García-Bayona et al., 2017; Xiong et al., 2018). The T6SS which is often known for its antagonism utilizes a bacteriophage-like subassembly to secrete effector proteins to both prokaryotic and eukaryotic target cells. The system shares both structural features as well as protein sequences with phages. In addition to the role of T6SS in mediating interbacterial competition, the system also has potential role in signaling and virulence (Hayes et al., 2010; Russell et al., 2014).

Interestingly, a contact-dependent inhibition mediated by glycine zipper proteins (Cdz) system involving a T1SS is reported in *C. crescentus* that enables the bacterium to kill the neighboring cells with contact-inhibition (García-Bayona et al., 2017).

### Contact-Dependent Mechanosensing

One of the important aspects of all the living cells is to sense and respond to various signals. Similarly, bacteria communicate with their environment through sensing and responding to chemical, biological and physical signals. In bacteria, surface-sensing and attachment dependent behavior indicate toward their physical-sensing or mechanosensing properties. These two steps are important and significant in the biofilm mode of bacterial growth. When a planktonic bacterium senses and reaches a surface to become sessile, it undergoes through various substantial changes. These involve sensing: (a) physicochemical changes, (b) attachment of cellular appendages, and then (c) attachment of the bacterial cell body to the surface (Kimkes and Heinemann, 2020). As the microenvironment near bacteria and surface is not same, difference is observed in them on the basis of ionic strength, nutrient availability, pH and osmolarity (Berne et al., 2018). To sense these differences, a two-component signal transduction system is usually used by bacteria. It comprises of a membrane-bound histidine kinase and a cytoplasmic response regulator to sense the stimulus and thus mediate the cellular response, respectively. For example in *E. coli* the major systems involved in sensing physicochemical changes and thus resulting in downstream regulation for biofilm formation are CpxAR, EnvZ/OmpR and RcsCDB (Gordon and Wang, 2019; Kimkes and Heinemann, 2020).

Next to this occurs the adhesion of bacterial cell appendages to the surface. Studies suggest that bacterial motility appendages such as type-IV pili, flagella and even the envelope proteins are likely to be the potential mechanosensory elements involved in adhesion (Gordon and Wang, 2019). As the bacterium attaches its appendages to the surface, due to the change in mechanical properties of bacterium’s environment, biological responses such as phenotypic changes are observed. Thus, with initial adhesion impairment in motility is observed and the movement of cell appendage is hindered (Stones and Krachler, 2016). This generates and transmits signals to the cell indicating its attachment to the surface.

Further adhesion of bacterial cell body to surface is mediated by interactions of various forces such as long-range Van der Waals, short-range repulsive electrostatic forces and acid-base interactions (Berne et al., 2018). This contact can be additionally supported by cell appendages or by the long O-antigen part of lipopolysaccharides (LPS). Moreover, production of adhesins also plays crucial role in attachment of cell body with surface in both polar and flat orientation. For example, *A. tumefaciens* and *C. crescentus* have polar orientation during attachment due to synthesis of polar adhesins at the cell poles whereas in *P. aeruginosa* with the production of an exopolymeric matrix component- Pel polysaccharide, a transition is observed from polar adhesion to a flat orientation. Following this entire process bacteria attaches to the surfaces, sticks together and promotes bacterial aggregation, colonization, and biofilm formation (Li et al., 2012; Cooley et al., 2013; Kimkes and Heinemann, 2020).

Studies related to contact-dependent signaling for communication conducted on various microorganisms such as on *E. coli*, *B. subtilis*, *M. xanthus*, and *Lactobacilli* suggests that physical contact apart from being a direct way of bacterial communication also helps in various mechanisms through which bacteria can optimize the use of quorum-sensing molecules. Thus physical contact mediated either through direct cell-to-cell contact or with the help of surface appendages helps in all the prevailing mechanism of bacterial communication (Harapanahalli et al., 2015).

As stated by Stacy and coworkers, that every biological interaction cannot be considered as genuine communication rather it can be a response to a cue or coercion or sometimes just a contact which eventually does not lead to any molecular exchange (Stacy et al., 2012). Thus, every contact developed through the surface appendages and cytoskeleton proteins do not necessarily lead to such communications in which both the emitter and receiver organism gains benefits but are actually contact-mediated mode of establishing connections that can eventually assist in communication or even in cue and coercion.

## Bacterial Cytoskeleton in Cell Communication

Besides surface appendages, bacterial cytoskeleton is also involved in cell communication. Interestingly, counterparts of all three-known eukaryotic cytoskeletal proteins (actin, tubulin, and Intermediate filaments) have been found in eubacteria that form filamentous structures and show cytoskeletal properties. In general, the three major functions carried out by cytoskeleton are: (1) spatially organizing the contents of the cells, (2) connects the cell to the external environment both biochemically and physically and (3) plays key role in cell motility, shape change and cell division. All these functions are performed by the dynamic and coordinated activities of the cytoskeletal proteins (Shih and Rothfield, 2006).

Interestingly, a study conducted on *Salmonella* with actin-like proteins, revealed that cytoskeleton proteins influence motility and colonization of the bacteria (Bulmer et al., 2012). In prokaryotes various actin-like proteins include MreB, MreB-like proteins (Mbl and MreBH), FtsA, ParM (StbA), ActA, etc. In *Salmonella*, MreB was found to be an essential protein. In Δ*mreC* mutant, the flagella system and the expression of virulence factors were found to be down regulated. Moreover, *mre* operon had shown to play an important role in colonization of the bacterium during infection. The study suggested the strong connection between the bacterial cytoskeleton and pathogenicity, as expression of virulence genes were observed to be in direct coordination with the cytoskeletal integrity (Bulmer et al., 2012).

Similarly, in *Helicobacter pylori* the key role of MreB cytoskeletal protein was to maintain the enzymatic activity of a virulence factor urease rather than maintaining cell shape (Waidner et al., 2009). Furthermore, a different aspect of the cytoskeleton protein MreB has also been demonstrated in *P. aeruginosa*. In this bacterium, MreB protein was found to be essential for the production and polar localization of type IV pili. The type IV pili are significant for virulence and even for providing antibiotic resistance to the bacterium by biofilm formation during chronic infections (Cowles and Gitai, 2010).

Motility is an important feature by which even far away cells can reach each other and communicate. Gliding motility is one of the motility type by which bacteria actively move over the surfaces without the involvement of flagella (McBride, 2001). *Myxococcus xanthus*, uses gliding motility to move along the solid surfaces even without the aid of type IV pili. But *M. xanthus* requires MreB, the bacterial actin for its motility. It has been observed that there is interdependency between the movement machineries of gliding motors and MreB filaments. This is analogous to the movement of myosin motors and actin in eukaryotic cells (Fu et al., 2018). Similar to this, gliding motility is observed in various species of mycoplasma such as in *M. genitalium*, *M. pulmonis*, and *M. mobile*, etc. A well-defined cytoskeleton comprising of 25 different proteins is reported in mycoplasma. It has been observed that the cytoskeleton of mycoplasma plays important role in its gliding motility as unusual or irregular shaped cells were observed in non-motile mutants of *M. mobile* (Miyata et al., 2000).

## Targetting Cell Communication

A report states that biofilm formation is involved in about two-thirds of the human infections. This includes infections of skin (integumentary), ears (auditory), urinary tract, respiratory tract, reproductive organs, digestive system, uncontrolled dental plaque, and fouling of various implants and even infection of contact lenses (Otto, 2019; Vestby et al., 2020). It becomes difficult to deal with such infections as biofilms provide more tolerance to bacteria against the antimicrobial treatments and even toward the host immune responses and defense mechanisms (Kimkes and Heinemann, 2019).

**FIGURE 4:**
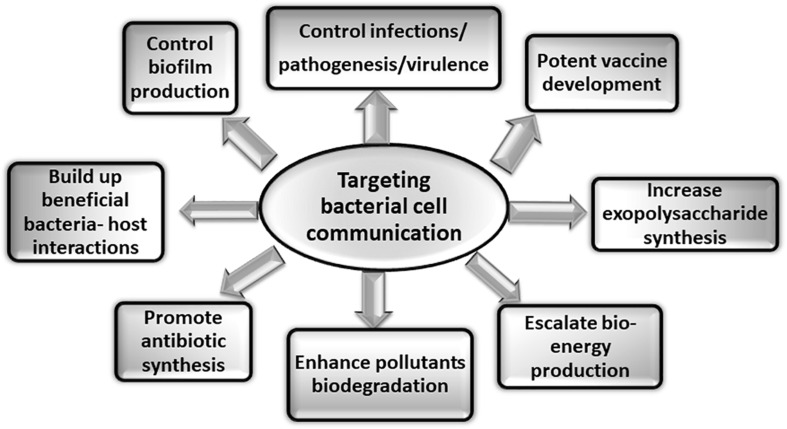
Applications of targeting bacterial cell communication. Studies targeting the various aspects of bacterial cell communication system are of immense importance. The figure shows some of the applications that can be explored by addressing bacterial communication systems.

The detailed understanding of the bacterial surface associated structures and related cytoskeleton proteins involved in cell communication will not only aid in designing of potential regulators or inhibitors in the form of novel therapeutics but will also have direct application in preventing biofilm formation one of the major causes of pathogenesis. Various other applications of targeting bacterial cell communication are illustrated in the Figure 4. There are various potential targets at different levels that can be explored for designing of therapeutics. On the basis of knowledge about biogenesis of various tubular extensions, the key genes responsible for the structural functionality of the surface appendages can be targeted. Production of exopolysaccharides and adhesive molecules responsible for irreversible attachment of pathogen to the host can also be blocked. There are various reports which demonstrate that even with blocking or hindering the flagellar rotation, biofilm formation can be controlled (Wood et al., 2006; Yoshihara et al., 2015). In a recent study conducted on *S. typhimurium* and *E. coli*, it was observed that the bacterial flagella strongly associated with the host cell membrane and disrupted it with flagellar rotation. It was seen that the bacterial flagella showed affinity toward actin and actin-binding proteins and binding was observed even during *in vitro* conditions. This phenomenon suggests the existence of molecular mechanism which connects both cytoskeletal dynamics and bacterial colonization (Wolfson et al., 2020). Vaccines against the building blocks of various apparatuses such as flagellin of flagella and pilin of pili can also be an alternative approach for preventing pathogenesis. As depletion of various cytoskeletal proteins have also shown to modulate the pathogenicity of many microorganisms either directly or indirectly, so identification of more such proteins and understanding their roles in pathogenic bacteria could be useful in hampering cell-to-cell contact, growth and ultimately survival of the pathogenic bacteria. Thus, targeting and blocking bacterial communication can be one of the most advanced way of preventing infectious diseases. Other advantageous aspects of targeting apparatuses involved in cell communication could be manipulating and monitoring biofilm formation for the betterment of mankind. Not all the biofilms are problematic whereas there are naturally occurring biofilms in the environment that biodegrades various pollutants, industrial effluents and also helps in waste water treatment (Bryers, 1990; Edwards and Kjellerup, 2013). So, by promoting cell communication in the respective microbial species *in situ* or *ex situ* (at the places where bioremediation is aimed), biofilm formation can be enhanced resulting in degradation of toxic compounds. This could be an approach for minimizing the build-up of pollutants at various places (Kumar and Anand, 1998). Extracellular polymeric substances (EPS) form the architecture of bacterial biofilm matrix. It provides various properties to the biofilm so as to shield and protect it from antibiotics and different forms of stress (Vu et al., 2009; Nwodo et al., 2012). Thus, it is primary attribute of the microbial community to produce EPS in order to exist in the form of biofilm. This property of the microbial cells can be exploited. Furthermore, the production of commercially important EPS, medicines, drugs, and antibiotics can be increased by implementing such techniques of controlled biofilm formation (Ortega-Morales et al., 2010). Moreover, similar to the healthy colonization of lactic acid bacteria (LAB) in the gastrointestinal tract, more such beneficial bacteria-host interactions can be established in the forms of consumable probiotics (Kumar and Anand, 1998). To support the tremendously increasing energy demand, production of bio-energy using micofluidic devices and microbial fuel cell is the current solution to the energy crises (Singh and Verma, 2015). This strategy can also explore the importance of microbial cell communication to address the issue more efficiently.

## Importance and Future Prospects

Currently, one of the major ongoing global concerns is the upsurge of anti-microbial resistance leading to the emergence of various antibiotic-resistant pathogens along with the enhanced rate of microbial evolution. In contrast to this, the development of novel antibiotics is lagging as it is being developed comparatively at a very slower rate. In this era of antibiotic-resistance, to combat with abruptly erupting deadly infectious diseases there is an urgent need of developing alternative strategies and innovative therapeutics (Njoroge and Sperandio, 2009). One such anti-virulence strategy is exploiting bacterial cell-to-cell communication. This approach could serve as a way of targeting and obstructing the outbreak of diseases. The bacterial pathogens cause harm not only to humans but equally affect other living forms such as animal health and agricultural productivity. These adverse effects of the bacterial pathogens can be controlled by hindering bacterial cell communication.

Such basic studies which aim to increase our knowledge about the bacterial systems and proteins involved in cell communication when combined with present-time modern technologies can definitely lead to development of various novel therapeutics. Research conducted on bacterial cytoskeletal system and other related apparatuses such as surface appendages which assist in communication are of great importance and hold immense expectations with their application in addressing issues related to bacterial infections and diseases.

## Author Contributions

DS and PS wrote and edited the manuscript.

## Conflict of Interest

The authors declare that the research was conducted in the absence of any commercial or financial relationships that could be construed as a potential conflict of interest.
